# Type IA Topoisomerases as Targets for Infectious Disease Treatments

**DOI:** 10.3390/microorganisms9010086

**Published:** 2021-01-01

**Authors:** Ahmed Seddek, Thirunavukkarasu Annamalai, Yuk-Ching Tse-Dinh

**Affiliations:** 1 Department of Chemistry and Biochemistry, Florida International University, Miami, FL 33199, USA; asedd001@fiu.edu (A.S.); athiruna@fiu.edu (T.A.); 2 Biomolecular Sciences Institute, Florida International University, Miami, FL 33199, USA

**Keywords:** topoisomerase, antimicrobial resistance, drug targets

## Abstract

Infectious diseases are one of the main causes of death all over the world, with antimicrobial resistance presenting a great challenge. New antibiotics need to be developed to provide therapeutic treatment options, requiring novel drug targets to be identified and pursued. DNA topoisomerases control the topology of DNA via DNA cleavage–rejoining coupled to DNA strand passage. The change in DNA topological features must be controlled in vital processes including DNA replication, transcription, and DNA repair. Type IIA topoisomerases are well established targets for antibiotics. In this review, type IA topoisomerases in bacteria are discussed as potential targets for new antibiotics. In certain bacterial pathogens, topoisomerase I is the only type IA topoisomerase present, which makes it a valuable antibiotic target. This review will summarize recent attempts that have been made to identify inhibitors of bacterial topoisomerase I as potential leads for antibiotics and use of these inhibitors as molecular probes in cellular studies. Crystal structures of inhibitor–enzyme complexes and more in-depth knowledge of their mechanisms of actions will help to establish the structure–activity relationship of potential drug leads and develop potent and selective therapeutics that can aid in combating the drug resistant bacterial infections that threaten public health.

## 1. Introduction

Infectious diseases are diseases where certain microorganisms grow and replicate inside a host, leading to damage or injury to body tissues of the host. Even normal flora can cause diseases to the host if the host is immunocompromised, for instance in cancer and acquired immunodeficiency syndrome (AIDS). Many effective antimicrobial agents have been discovered and marketed so far. However, there has been a constant issue that counteracts the effects of well-established antimicrobial agents, i.e., antimicrobial drug resistance [1,2,3].

Antimicrobial resistance has been a major global health concern in recent years. Mechanisms of resistance vary [4,5], such as degrading or changing the drug molecule to an inactive form inside the microorganism [6], modifying or protecting the target for the antimicrobial agent by the microorganism [7,8], or pumping out the drug molecules from the microorganism cell, known as efflux [9,10]. Resistance to antimicrobial agents is usually genetically encoded by the microorganism, either on the bacterial chromosome, or on a plasmid that can be spread among different strains of microorganisms, resulting in the emergence of new resistant strains [11,12].

The number of deaths attributable to antimicrobial resistance is expected to continue growing, hitting around 10 million cases in 2050 [13]. Worldwide, tuberculosis (TB) is one of the top 10 causes of death. According to the WHO, TB is the leading cause of death from a single infectious agent. TB caused an estimated 1.4 million deaths worldwide in 2019, with an estimated 10.0 million new cases of TB, equivalent to 130 cases per 100,000 population [14]. Globally, there were an estimated 558,000 new cases of rifampicin-resistant TB (RR-TB). Among RR-TB cases, an estimated 82% were multidrug-resistant TB (MDR-TB). Furthermore, 3.5% of new TB cases and 18% of previously treated cases were MDR/RR-TB. Drug resistant TB is very difficult to treat [15]. Other examples of drug resistant bacteria include the *Enterococcus faecium*, *Staphylococcus aureus*, *Klebsiella pneumoniae*, *Acinetobacter baumannii*, *Pseudomonas aeruginosa* and *Enterobacter* spp. (ESKAPE) pathogens [16,17].

Following the discovery of daptomycin in late 1980s [18], the process of novel antibiotic discovery stagnated and could not keep up with the emergence of antibiotic resistant pathogens [19,20]. Hence, the discovery of novel antimicrobial agents is an urgent need. DNA topoisomerases are well known targets for antibacterial drug discovery [7,21,22,23,24,25]. Topoisomerases are responsible for controlling the topology of the genome, including local and global DNA supercoiling [26,27]. DNA topology and topoisomerase functions play important roles in replication, transcription, and genome stability [21,27,28,29,30,31,32].

In general, all DNA topoisomerases work by binding to the target DNA molecule, cleaving one or both strands of the double helix, passing another single or double strand through the break, and resealing the DNA [21,30]. A covalent complex is transiently formed between an active tyrosine residue of the topoisomerase enzyme and one end of the broken strand during the cleavage process. [33]. The covalent complex with cleaved DNA is a vulnerable intermediate that can lead to cell death if trapped.

The interests in studying the structures, functions, mechanisms of actions, and inhibitors of DNA topoisomerases are mainly due to two reasons: (1) the pivotal role of DNA topoisomerases in the management and control of DNA topology [21,30,32] and (2) development of topoisomerase inhibitors into widely used clinical therapies for cancer and bacterial infections [21,24,25,34,35,36]. Many efforts have been exerted so far to either synthesize new molecules, or discover new bioactive molecules, which could potentially inhibit specific members of the DNA topoisomerase family, to provide new treatment for cancer and infectious diseases.

## 2. Type IA Topoisomerases

DNA topoisomerases are divided into two main categories: type I topoisomerases and type II topoisomerases. Differences in DNA strand passage activity and catalysis are the basis of this categorization, and each main category is subdivided into subcategories according to the mode of DNA binding and relaxation. Type I topoisomerases break and rejoin a single DNA strand during catalysis. On the other hand, type II topoisomerases break and rejoin the two strands of a double helix in order to pass another intact double helix through the break, promoting catenation/decatenation, unknotting/knotting, and relaxation of positively and negatively supercoiled DNA [37]. Type II topoisomerases are subcategorized into two subfamilies: topoisomerases IIA and topoisomerases IIB. All type II topoisomerases covalently attach to the 5′ end of the DNA through a phosphotyrosine bond, requiring divalent magnesium cation and ATP hydrolysis. Archaeal and bacterial type IIA topoisomerases, such as *Escherichia coli* DNA gyrase, are A_2_B_2_ heterotetramers. In contrast, eukaryotic type IIA topoisomerases are all homodimeric enzymes. The discovery of topoisomerase VI, an A_2_B_2_ heterotetrameric type II topoisomerase found in all archaea, added a new type IIB subfamily to the type II topoisomerases. The relative arrangement of elements required for type II topoisomerase’s mechanism as well as the overall structural organization are dissimilar between the type IIA and type IIB topoisomerases [30]. Type IIA topoisomerases cleave the opposing strands of duplex DNA with a four-base stagger, while two-base staggered cuts are made by type IIB topoisomerases [37].

**Figure 1:**
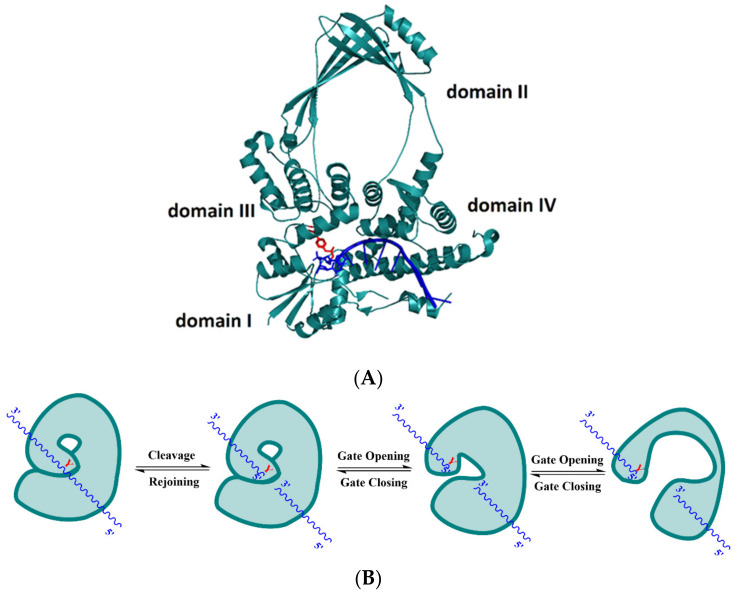
DNA-gate formed by cleavage of G-strand DNA by type IA topoisomerase (**A**) Crystal structure of covalent complex between single-stranded DNA (ssDNA) and *E. coli* topoisomerase I *N*-terminal domains I to IV (Protein Data Bank (PDB) 3PX7). The active tyrosine residue Y319 is illustrated with the red color, and ssDNA is represented by the blue color. (**B**) Gate opening and closing dynamics of bacterial topoisomerase IA. The enzyme is represented by the green shape and the ssDNA is represented by the blue wavy line. The letter Y stands for the active site tyrosine residue Y319.

Type I topoisomerases are subcategorized into three subfamilies: topoisomerases IA, topoisomerases IB, and topoisomerases IC [38]. Members of the topoisomerase IA subfamily share common mechanistic features, such as being monomeric proteins and covalent attachment to the cleaved DNA end through a 5’ phosphotyrosine bond. Divalent cation is needed as a cofactor to complete the catalytic process. Finally, the members of this subfamily can relax negative supercoils because they only require single stranded regions of the target DNA molecule for binding [39]. In the protein-mediated DNA gate model for strand passage by type IA topoisomerases [40,41], a segment of single-stranded DNA (ssDNA) binds through domains I and IV, and the active tyrosine residue in domain III performs the nucleophilic attack on this G-strand ssDNA to create a break. Movement of domains I and III away from each other results in gate opening (Figure 1) to allow passage of another ssDNA strand (T-strand). Gate-opening dynamics of *E. coli* topoisomerase I and III were observed recently in single-molecule studies [42]. The presence of Mg^2+^ divalent ions is necessary for the process of DNA rejoining by type IA topoisomerases [43,44,45]. The divalent ion is hypothesized to help position the 3′-hydroxyl group of the cleaved DNA in proximity to the phosphotyrosine linkage, where the 3′-hydroxyl group performs a nucleophilic attack on the phosphotyrosine linkage leading to rejoining of the DNA backbone [46]. The requirement for divalent ions and formation of 5-phosphotyrosine bond are mechanistic similarities between type IA and type IIA topoisomerase. In contrast, type IB and type IC topoisomerases do not require divalent ions for catalysis and are linked to the 3′-phosphate in the covalent intermediate. There is no sequence or structural homology between type IA, type IB and type IC topoisomerases [47]. Type IA topoisomerases are the only family of topoisomerases that can catalyze topological changes on both DNA and RNA molecules [48]. The activity of type IA topoisomerases is needed for overcoming DNA or RNA topological barriers that require strand passage through the break mediated by the topoisomerase IA on a single strand of nucleic acid [32,49]. At least one type IA topoisomerase is present in all free-living organisms [50] found in the three kingdoms of life. Drugs targeting bacterial or parasitic type IA topoisomerases for treatment of infectious diseases must be selective in not inhibiting the human type IA topoisomerases TOP3A and TOP3B.

### 2.1. Rationale for Type IA Topoisomerases as Drug Targets

Since topoisomerases play key roles in managing the topology of DNA, which, in turn, is a major factor impacting cell viability, they have been widely targeted for clinical treatment of cancer and infectious diseases [24,25,51]. Topoisomerase inhibitors can be classified based on the mechanism of action as poison inhibitors or catalytic inhibitors [52,53]. Poison inhibitors are agents that work by stabilizing the topoisomerase covalent complex that is formed as an intermediate during catalysis, while catalytic inhibitors are agents that work by interfering with the catalytic cycle of the enzyme in any other step, such as inhibitors that prevent initial substrate binding or formation of the cleavage complex. Topoisomerase targeting drugs in current clinical use are poison inhibitors against type IB and type IIA topoisomerase [52,53,54]. Topoisomerase poison inhibitors cause loss of cellular viability through accumulation of breaks in the chromosome, triggering apoptosis in cancer cells [55] and oxidative damage cell death pathway in bacteria [56]. This mechanism of cell death is highly efficient since it does not require the activity of most of the target topoisomerase molecules to be blocked and the topoisomerase activity does not need to be essential. Even though there are currently no approved drugs that utilize type IA topoisomerases as cellular targets, it can be argued that since all topoisomerases utilize an active site tyrosine to form the covalent intermediate, it should also be possible for a poison inhibitor to trap the covalent complex formed by type IA topoisomerases.

### 2.2. Validation for Bacterial Topoisomerase IA as a Novel Antibiotics Target

Topoisomerase I and topoisomerase III are type IA topoisomerases that are present in bacteria, with reverse gyrase being found in thermophilic bacteria. *E. coli* has both topoisomerase I and topoisomerase III present. Bacterial topoisomerase I is much more efficient for relaxation of negatively supercoiled DNA during transcription while topoisomerase III has a stronger decatenation activity for resolution of replication and recombination intermediates [57,58,59,60]. Null mutation of *topA* in *E. coli* can be compensated by mutations that reduce the activity of gyrase that has the opposite effect on DNA supercoiling [61,62,63]. Nevertheless, endogenous inhibitors of topoisomerase I activity including overexpressed Tn5 transposase [64,65], T4 gp55.2 [66] and toxin YjhX [67] can inhibit cell growth and result in a loss of viability. Furthermore, even though the essentiality of topoisomerase I in some bacteria is uncertain, poison inhibitors against the type IA topoisomerase present in every bacterium should generate bactericidal DNA lesions. The bactericidal consequence of topoisomerase I covalent complex accumulation in *E. coli* was demonstrated through identification of bacterial topoisomerase I mutants deficient in DNA rejoining [44,45,68,69,70]. The mutations involved include mutations in the conserved D111, D113 and E115 (DDE) triad that bind Mg^2+^ required for DNA religation [69]. These dominant lethal topoisomerase I mutations mimic the expected action of type IA topoisomerase poison inhibitors. It should also be noted that YjhX overexpression resulted in *E. coli* cell death, even though YjhX has been shown not to be a poison inhibitor of *E. coli* topoisomerase I [67]. Therefore, inhibitors of bacterial topoisomerase I do not necessarily have to act as topoisomerase poisons to be bactericidal.

Studies have shown that topoisomerase I is essential for cellular viability of a number of important bacterial pathogens, including *Mycobacterium tuberculosis* [71,72], *Streptococcus pneumoniae* [73], *P. aeruginosa* [74] and *Helicobacter pylori* [75]. In bacteria that only have one type IA topoisomerase, the catalytic activity of the enzyme should be essential for overcoming topological barriers that require passage of one strand of DNA through another single strand [32], and catalytic inhibitors of topoisomerase I would have antibacterial efficacy regardless of the mechanism of topoisomerase inhibition. Hence, bacterial topoisomerase IA represents a valid target for novel antibiotics aimed at overcoming antimicrobial resistance.

### 2.3. Eukaryotic Type IA Topoisomerases as Potential Target for Infectious Disease Treatment

Fungi have only one type IA topoisomerase, TOP3, that has high degree of similarity to bacterial topoisomerase III. The TOP3 mutant is not viable in *Saccharomyces pombe* [76,77] and has a slow growth phenotype in *Saccharomyces cerevisiae* [78]. In higher eukaryotes, the TOP3 gene has been duplicated into TOP3A and TOP3B isoforms [50]. TOP3A is essential for mice embryo development [79]. TOP3B knockout mice developed to maturity but had a shortened life span [80]. Human topoisomerase III beta (TOP3B) has been shown to have both DNA and RNA topoisomerase activities [81,82]. It relaxes hyper-negatively supercoiled DNA in transcription and prevents R-loop accumulation [83,84]. Interaction between TOP3B and mRNAs [85] is required for neurodevelopment and synapse formation [81,82]. Human TOP3B is stabilized by Tudor domain containing 3 protein (TDRD3) [84,86,87], which was previously identified as a host protein needed for efficient replication of flaviviruses, such as dengue and yellow fever virus [88]. However, subsequent follow-up studies on the role of TDRD3 in viral replication have revealed that TOP3B is the actual host factor required for the efficient replication of all positive-sense single-stranded RNA viruses, with TDRD3 playing a role in the stabilization of TOP3B [49]. Specific inhibitors for TOP3B could potentially be useful as broad-spectrum antiviral treatments against flaviviruses and coronaviruses including SARS COV-2 [49].

Topoisomerases in kinetoplastid parasites are potential targets for the development of improved therapeutic options against deadly parasitic diseases [89,90]. Three type IA topoisomerases are present in the kinetoplastids. In addition to functionally active TOP3A [91] and TOP3B [92], a prokaryotic-like mitochondrial TOP1A is also present. The mitochondrial TOP1A in *Trypanosoma brucei* is essential for late theta replication intermediates structure resolution [93]. Since the mitochondrial TOP1A has no close homologs in humans, it could be a target for selectively toxic new antiprotozoal treatments [93].

## 3. Screening Approaches

### 3.1. In Silico Screening

Several strategies and techniques can be used to identify novel topoisomerase inhibitors for treatment of infectious diseases. A commonly used strategy is screening chemical libraries composed of a large number of molecules for the desired activity. This strategy has been widely used for decades in drug discovery. The chemical libraries used could comprise of synthetic compounds, natural products, or both. In silico virtual screening employing docking and molecular dynamics (MD) simulations can be used first to identify potential inhibitors from compound libraries, followed by biochemical assays on a small number of in silico hits with the highest predicted binding affinity to confirm the interaction and assess the topoisomerase inhibition and antimicrobial activity. This approach has been applied to identify inhibitors for *E. coli* and *M. tuberculosis* topoisomerase I using either crystal structures or target structures generated by computational modeling [94,95,96,97,98].

### 3.2. Biochemical Screening Assays

Various biochemical assays can be used to assess the ability of a compound to inhibit the activity of a specific topoisomerase, and to determine the mechanism of inhibition so that the hit can be classified either as a catalytic inhibitor or as a poison *inhibitor*. Examples include, but are not limited to, gel based-assays, fluorescence-based assays, and detection of DNA-enzyme covalent complex via the rapid approach to DNA adduct recovery (RADAR) assay.

Agarose gel electrophoresis can be used to determine the inhibition of the bacterial topoisomerase I, since this enzyme relaxes supercoiled plasmid DNA. A supercoiled plasmid DNA, representing inhibited enzyme or control reaction without enzyme, will migrate the fastest, while the fully relaxed plasmid DNA will migrate the slowest in an agarose gel electrophoresis experiment [99]. DNA can be visualized using ethidium bromide staining. The presence of an effective inhibitor will prevent the enzyme from relaxing the plasmid DNA, and it will instead remain supercoiled until the concentration of the inhibitor is sufficiently low for the enzyme to regain its activity. To further determine the mode of inhibition, single-stranded DNA or oligonucleotide substrate labeled with ^32^P at the 5′-end can be used to visualize the shortened cleavage product that results from topoisomerase I cleaving the substrate following electrophoresis of the reaction products in denaturing acrylamide gel [100]. A topoisomerase poison inhibitor is expected to result in the increase in accumulated cleavage product.

Since gel electrophoresis is time consuming and low in throughput capacity, several fluorescence-based assays have been developed to monitor topoisomerase activities and detect potential inhibitors with high throughput capacity. One such assay involved the use of an immobilized triplex-forming oligonucleotide capable of selectively capturing supercoiled plasmids that can be quantified by SYBR Green dye [101]. Another group of researchers have developed a homogenous assay that measures the fluorescence anisotropy of the triplex-forming oligonucleotide and can be applied to assay the activity of both *E. coli* gyrase and topoisomerase I [102]. A different approach is to insert into a plasmid an inverted repeat containing a fluorophore-quencher pair that can emit fluorescence depending on whether cruciform formation is facilitated by negative supercoiling of the plasmid [103,104]. An assay based on supercoiling-dependent fluorescence quenching has been used to study the kinetics of relaxation of negatively supercoiled DNA by *E. coli* and *Mycobacterium smegmatis* topoisomerase I [105]. However, these fluorescence-based assays designed for the measurement of supercoiling/relaxation of plasmid DNA have not yet been applied to screen for bacterial topoisomerase I inhibitors from a large compound library. An oligonucleotide substrate with fluorophore and quencher at the 5′ and 3′ ends of the stem-loop structure was designed to detect the inhibition of DNA religation by topoisomerase I poison inhibitors [106]. This assay identified organomercury compounds that act as poison inhibitors against *E. coli* and *Y. pestis* topoisomerase I that bind Zn(II) with cysteines [100].

The rapid approach to DNA adduct recovery (“RADAR”) assay was first developed to purify and measure the level of topoisomerase–DNA complex trapped by anticancer drugs that act as topoisomerase poison inhibitors in mammalian cells [107]. A modified RADAR/ELISA assay can also measure the level of gyrase covalent complex trapped by fluoroquinolones in bacterial cells [108]. Recently, it has been shown that this RADAR/ELISA assay can detect in *E. coli* and *M. smegmatis* increased accumulation of covalent complex formed by bacterial topoisomerase I with mutations that inhibit DNA rejoining, providing a potential assay for the discovery and optimization of drugs that act as bacterial topoisomerase I poison inhibitors [109].

## 4. Recent Attempts to Discover Novel Bacterial Topoisomerase I Inhibitors

During the past decade, various attempts have been made to find inhibitors of bacterial topoisomerase I to provide potential leads for the development of novel antibiotics, aiming to combat antimicrobial resistance. These inhibitors are discussed below and the half maximal inhibitory concentrations (IC_50_s) for inhibition of *E. coli* or *M. tuberculosis* topoisomerase I activity are shown in Table 1.

### 4.1. Bis-Benzimidazoles

**Figure 2:**
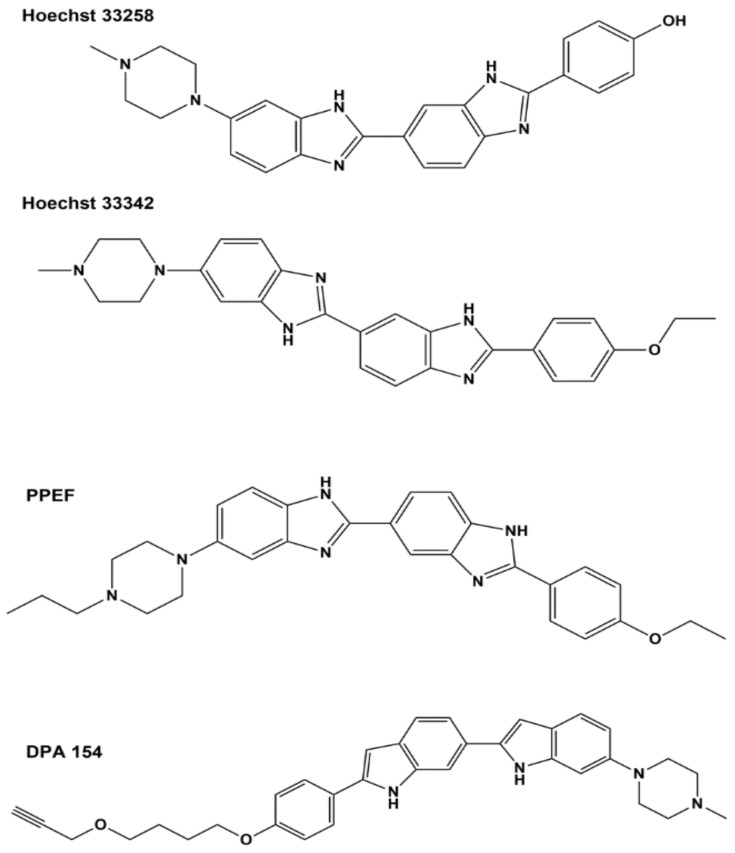
Structures of bis-benzimidazoles Hoechst 33258, Hoechst 33342, 2′-(4-ethoxyphenyl)-5-(4-propylpiperazin-1-yl)-1H,1′H-2,5′-bibenzo[*d*]imidazole (PPEF) and DPA 154.

Bis-benzimidazole derivatives have gained much attention as potential therapeutics since the development of Hoechst bis-benzimidazole 33,258 and 33,342 as DNA minor groove binders that inhibit human topoisomerase I and show activity against certain types of cancers [117,118], but the drug development efforts did not progress to clinical trials due to high cytotoxicity [119]. A series of bis-benzimidazoles with closely related structures were synthesized by two different groups and found to provide selective inhibition of type IA *E. coli* topoisomerase I over inhibition of type IB human topoisomerase I [110,120]. The bis-benzimidazoles specific for *E. coli* topoisomerase I exhibited low minimum inhibitory concentrations (MICs) of 8 µg/mL against bacterial pathogens that included methicillin-resistant *S. aureus (MRSA)*, vancomycin-resistant *Enterococcus faecalis*, *Staphylococcus epidermidis*, *A. baumannii, Shigella flexneri* and clinical isolates of *E. coli* [110,111,121]. However, most of these derivatives still had cytotoxicity concerns, due to their DNA-binding activity [110,121]. DPA 154, shown in Figure 2, was also reported to elicit changes in the cellular ultrastructure including induction of spheroplasts and membrane lysis, suggesting a dual mechanism of antibacterial action that includes compromising cell membrane integrity [122]. In vivo efficacy against *E. coli* was demonstrated in the mouse systemic infection model as well as the mouse neutropenic thigh model [111] for one of the bis-benzimidazoles, 2′-(4-ethoxyphenyl)-5-(4-propylpiperazin-1-yl)-1H,1′H-2,5′-bibenzo[*d*]imidazole (PPEF), shown in Figure 2. It was proposed that PPEF shifts the cleavage–religation equilibrium of *E. coli* topoisomerase I by binding to the strictly conserved acidic triad D111, D113 and E115 (DDE) based on results from a site-directed mutagenesis study [123] where the aspartate and glutamate residues in the acidic triad were replaced with alanine residues. Circular dichroism studies and in silico docking have shown reduced binding of PPEF to these mutants, especially for the double mutants where two out of the three DDE residues were replaced by alanine [123]. This acidic triad of bacterial topoisomerase I is known to be involved in interactions with Mg^2+^ ions [124,125]. Substitution of the first aspartate to asparagine has been shown to affect metal binding and result in deficiency of DNA religation [69].

### 4.2. Tricyclic Antidepressants

**Figure 3:**
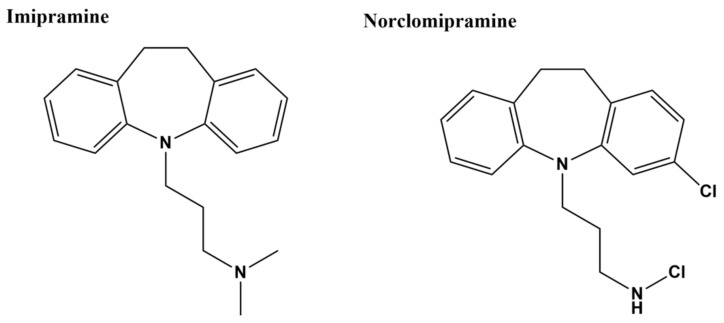
Structures of tricyclic antidepressant imipramine and metabolite norclomipramine.

In silico docking was conducted to screen a library of FDA-approved drugs for their potential activity against a homolog model of *M. tuberculosis* topoisomerase I with Mg^2+^ and a DNA fragment bound in the open state [96]. Clinically prescribed tricyclic antidepressant imipramine and norclomipramine, which is the active metabolite of another tricyclic antidepressant (clomipramine), were then selected based on the docking results for biochemical and whole cell assays. Imipramine and norclomipramine (Figure 3) were found to inhibit *M. tuberculosis* topoisomerase I relaxation activity at <0.1 µM concentration following 15 min preincubation with the enzyme [96]. However, inhibition of *E. coli* topoisomerase I was not detected at up to 25 µM compound concentration. In silico docking with the *M. tuberculosis* topoisomerase I homology model has placed imipramine and norclomipramine near the strictly conserved DDE acidic triad. Alanine substitution at the glutamate in the third position abolished inhibition by the compounds while alanine substitution at the first aspartate had no effect on the inhibition. Both compounds showed a slight increase in cleavage product from a ^32^P-labeled double stranded oligonucleotide substrate and enhanced cytotoxicity in *M. smegmatis* overexpressing recombinant mycobacterial topoisomerase I, consistent with the compounds acting with a poison inhibitor mechanism [96]. However, the MICs required to stop the growth of *M. tuberculosis* were relatively high (60 µM for norclomipramine and 250 µM for imipramine). The tricyclic antidepressants have hard-to-tolerate side effects and several drug–drug interactions, which have recently led to the clinical preference of other classes of antidepressants [126]. Therefore, the tricyclic antidepressants are probably not a practical clinical option for targeting bacterial topoisomerase I.

### 4.3. Inhibitors Based on Polyamine Scaffold

**Figure 4:**
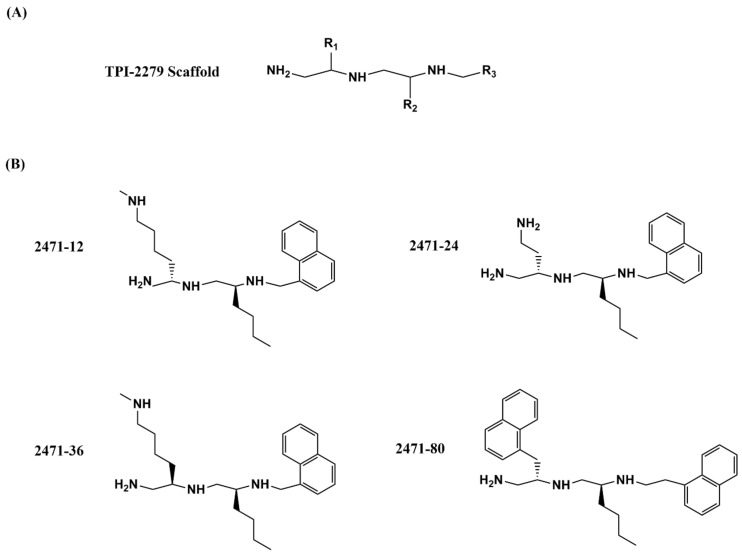
(**A**) Polyamine scaffold and (**B**) individual polyamine inhibitors of bacterial topoisomerase I with antimycobacterial activity [112].

The collection of small molecules at Torrey Pines Institute (TPI) now consists of more than 80 million small molecules organized into a small number of scaffold-ranking libraries so that promising molecular scaffolds can be identified quickly from a positive result in a biological assay. Structural analogs in the selected scaffold library mixtures can be studied with position scanning to identify the most favorable substitutions in the specific molecular scaffold [127,128,129]. This approach has been applied previously for the discovery of bacterial tyrosine recombinase and site-specific recombinase inhibitors [130]. In efforts to discover small molecules targeting bacterial topoisomerase I, inhibition of *E. coli* topoisomerase I relaxation activity was assayed first to identify the most promising scaffold and then the most inhibitory substitutions at three different R positions of the selected polyamine scaffold (Figure 4A). Fourteen related compounds that were synthesized as part of the 2471 series based on the position scanning data showed IC_50_ values < 20 µM against *E. coli* and *M. tuberculosis* topoisomerase I, and MIC values < 50 µM against *M. smegmatis* [112]. Among these 14 compounds, four polyamine analogues (Figure 4B) were confirmed to be also active against *M. tuberculosis* with selectivity index (50% cytotoxic concentration (CC_50_) against J774 macrophage cell line/IC_50_ against *M. tuberculosis*) values of up to 15. The observed inhibition of bacterial type IA topoisomerases by the 2471 compounds is unlikely due to non-specific inhibition of the type IA topoisomerase activity by the positively charged polyamine structure, or non-specific binding of the polyamine inhibitors to DNA, as the IC_50_ values for inhibition of human topoisomerase IB and topoisomerase II activities were >10 fold higher. Viable colony counts obtained following treatment of *M. smegmatis* and *M. tuberculosis* showed that both MICs and cell killing efficacies of these four polyamine analogs are sensitive to the level of topoisomerase IA activity. The bacteria became less sensitive when recombinant *M. tuberculosis* topoisomerase I was overexpressed, indicating catalytic inhibition of the topoisomerase IA activity as part of the antibacterial mechanism of action. Further improvement of potency and selectivity is desirable for this class of type IA topoisomerase inhibitors.

### 4.4. Gold(III) Complexes

**Figure 5:**
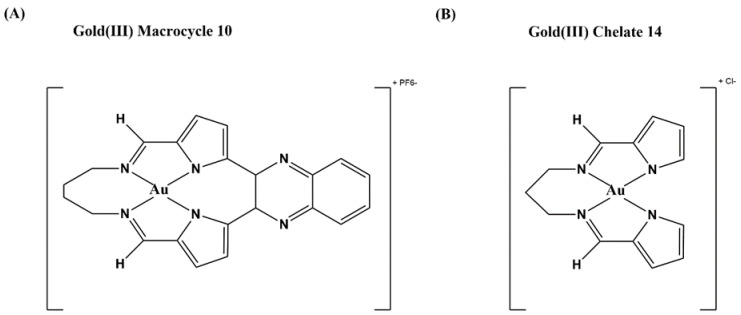
Gold(III) inhibitors of bacterial topoisomerase I [113]. (**A**) Gold(III) macrocycle 10 and (**B**) gold(III) chelate 14.

Metal-containing complexes have been used frequently as therapeutics throughout history, such as the well-known anti-cancer drug cisplatin [131] and the gold-containing auranofin used to treat rheumatoid arthritis [132]. Gold(III) tetra-aryl porphyrins with anticancer activity were found to bind DNA and inhibit human topoisomerase I [133]. That led to an increased interest in using gold compounds to inhibit topoisomerases. A number of pyrrole-based gold(III) macrocycles were synthesized and tested for anticancer activity and inhibition of human topoisomerase I [134]. The gold(III) macrocycle 10 illustrated in Figure 5A was found to be a catalytic inhibitor of human topoisomerase I with selectivity over human topoisomerase IIA. Gold(III) macrocycle 10 also caused lethality to many of the NCI’s panel of 60 human cancer cell lines [134]. In a follow up study investigating potential antibacterial application, some of the gold(III) macrocycles and additional bis(pyrrolide-imine) gold(III) chelates were found to inhibit the growth of both *M. tuberculosis* and *Mycobacterium abscessus*. The gold(III) chelate 14 (Figure 5B) showed a MIC of 1 µM against *M. tuberculosis* and 10 µM against *M. abscessus*, with bactericidal activity against mycobacteria as well. Gold(III) macrocycle 10 had even lower MICs against the mycobacteria but was not bactericidal, so these two compounds may have differences in their mechanism of action. Gold(III) chelate 14 showed no activity against Gram-negative species and only low level inhibition against other Gram-positives, suggesting that it has a specific activity against mycobacteria, including fluoroquinolone-resistant strains [113]. Both compounds shown in Figure 5 inhibit the relaxation activity of *M. tuberculosis* and *E. coli* topoisomerase I (Table 1), without any effect on bacterial DNA gyrase activity, suggesting the specificity these two compounds have for bacterial type IA topoisomerases inhibition [113]. Further studies are required to characterize the mechanism of antimycobacterial action of these gold(III) compounds and provide more insights in their structure–activity relationship (SAR) to limit the cytotoxicity.

### 4.5. Fluoroquinophenoxazine Derivatives

**Figure 6:**
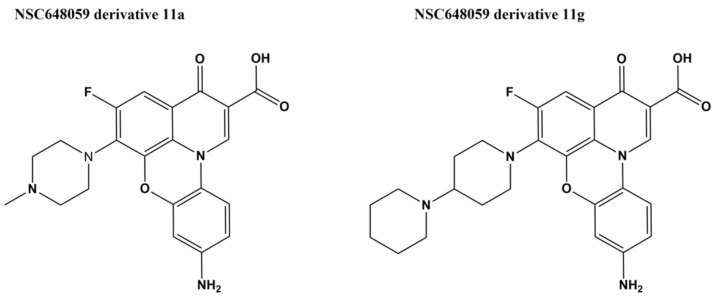
Structures of fluoroquinophenoxazine 11a and 11g [114].

A fluoroquinophenoxazine NSC648059 was initially identified in enzyme-based screening of *E. coli* topoisomerase I inhibitors [114]. A series of fluoroquinophenoxazine derivatives were synthesized to evaluate the SAR and to probe the structural elements of the fluoroquinophenoxazine core towards type IA topoisomerase target recognition. These derivatives were assayed for selectivity in inhibition of type IA topoisomerases, and antibacterial activity. Comparative molecular field analysis (CoMFA) of the three-dimensional quantitative structure–activity relationship (3D-QSAR) of these fluoroquinophenoxazine derivatives resulted in a model with reasonable statistics (*q*^2^ = 0.688 and *r*^2^ = 0.806) and predictive power (predictive correlation coefficient r^2^_pred_ = 0.767). While the structures of these fluoroquinophenoxazines have similarities to fluoroquinolones that target bacterial type IIA topoisomerases, this series of fluoroquinophenoxazines tested for selectivity exhibited much greater potency for inhibition of the type IA bacterial topoisomerase than inhibition of *E. coli* gyrase, human type topoisomerase I and IIα [114]. Derivative 11a with 6-methylpiperazinyl and 9-amino groups (Figure 6, IC_50_ = 0.48 µM) showed broad spectrum antibacterial activity (MICs = 0.78–7.6 µM) against bacteria that include Gram-negative *E. coli* and *M. tuberculosis*. Derivative 11g with the 6-bipiperidinyl lipophilic substitution showed the most promising antitubercular activity (MIC = 2.5 µM, selectivity index (SI) = 9.8) and was also active (MIC = 50 µM) against a clinical isolate of *Mycobacterium abscessus* [114,115]. The mechanism of resistance in mycobacteria was investigated by stepwise isolation of resistant mutants in *M. smegmatis* followed by comparison of the whole-genome sequence of the original strain and mutant isolates. Mutations in genes that affect compound entry and retention were identified instead of mutations in the topoisomerase I gene that might confirm the enzyme as the antibacterial target [115]. Biophysical analysis showed that DNA binding by fluoroquinophenoxazine 11g may contribute to the antibacterial mechanism, but could not account entirely for the direct binding and potent inhibition of the mycobacterial topoisomerase I [115].

### 4.6. Vichem’s Benzo(g)quinoxaline Compound

**Figure 7:**
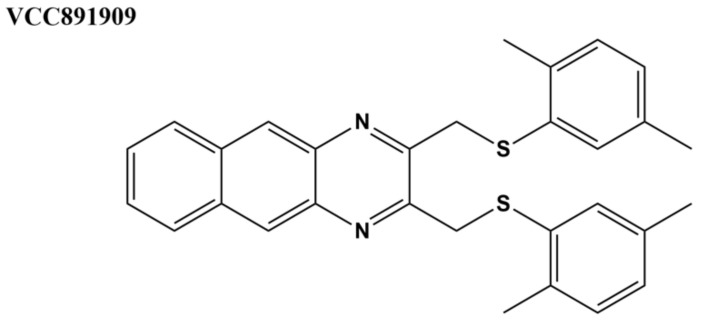
VCC891909 from Vichem’s Nested Chemical Library (NCL) [98].

Compounds with antitubercular activity were identified with whole-cell assays from approximately 17,000 molecules in Vichem’s Nested Chemical Library (NCL) [135]. The 639 compounds with MIC values < 6 μM were assayed for inhibition of *M. tuberculosis* topoisomerase I activity, and the results on the 108 compounds found to be active at 100 μM were used for building machine learning models for *M. tuberculosis* topoisomerase I inhibitors [97]. A selected set of seven Vichem NCL molecules with benzo(g)-quinoxaline, quinoxaline, or styryl-benzo(g)-quinazoline scaffolds was further characterized [98]. Cytotoxicity was measured against three human cell lines. In silico docking predicted strong binding affinity to specific sites on *M. tuberculosis* topoisomerase I through interactions with R167 and R114 [97,98]. The interaction of the selected hits with the human ATP-binding cassette (ABC) multi-drug transporters was assessed for potential drug resistance. The human ABCB (MDR/TAP) transporter family plays a major role in multiple drug resistance (MDR) against a variety of tuberculosis therapies, such as fluoroquinolones and aminoglycosides [98]. Most of the hits demonstrated considerable interaction with the transporters, except for VCC891909 (Figure 7). This compound also showed no cytotoxicity in all the human cells lines tested in the cytotoxicity assays with efficient inhibition of *M. tuberculosis* growth (MIC_90_ = 10.4 µM against H37Rv) and topoisomerase I activity (complete inhibition at 7.5–10 µM), suggesting that VCC891909 might be a good candidate for further antimycobacterial drug development [98].

### 4.7. Alkaloids SCN and N-SCN

**Figure 8:**
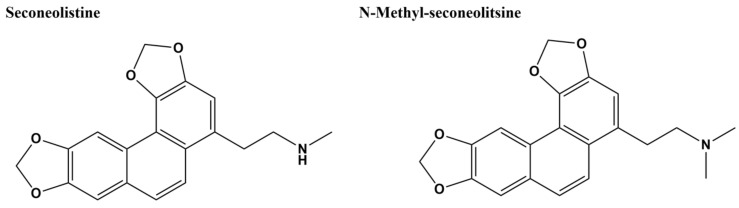
Alkaloids seconeolitsine (SCN) and *N*-methyl seconeolitsine (*N*-SCN) [136].

Two natural products and boldine-derivative alkaloids, seconeolitsine (SCN) and *N*-methyl-seconeolitsine (*N*-SCN) (Figure 8), were previously found to inhibit both topoisomerase I activity and cell growth of *Streptococcus pneumoniae* at approximately 17 µM [136]. The targeting of topoisomerase I by these compounds was supported by evidence of hypernegative supercoiling of DNA in treated cells along with protection from overexpression of topoisomerase I [136]. SCN has been used to demonstrate the homeostatic regulation of DNA supercoiling in *S. pneumoniae* by the topoisomerase I gene and the transcriptomic response to changes in local and global supercoiling [137,138]. These two alkaloids were tested more recently as potential inhibitors of *M. tuberculosis* topoisomerase I [116]. SCN and *N*-SCN exhibited MIC values below 16 µM against multiple isolates of *M. tuberculosis* and low IC_50_ values against the *M. tuberculosis* topoisomerase I (Table 1). SCN was shown to inhibit the cleavage of a 5′-biotin-labeled 32-mer oligonucleotide by the enzyme. The impact of *N-*SCN on DNA relaxation by topoisomerase I was confirmed by results from the analysis of topoisomer distribution of the mycobacterial plasmid by two-dimensional agarose gel, showing that the compound induced a 20% increase in plasmid supercoiling [116]. The action of both SCN and *N*-SCN as topoisomerase I catalytic inhibitors instead of poison inhibitors I was further confirmed by the decreasing MIC values of both compounds against *M. smegmatis* strain that expresses decreased levels of topoisomerase I [116]. Partial inhibition of human topoisomerase I could be observed at 50 µM concentration of SCN and *N*-SCN, with a small but significant effect on the viability of neutrophils at 100 µM concentration [136].

### 4.8. Additional Small Molecule Inhibitors Identified from Virtual Screening

**Figure 9:**
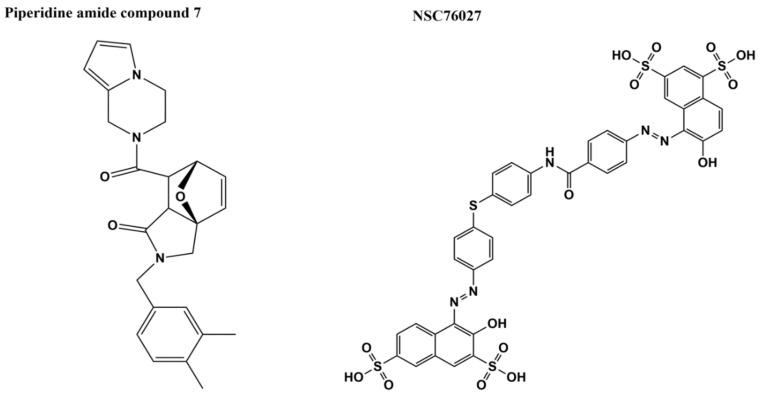
Bacterial topoisomerase I inhibitors identified by in silico screening—piperidine amide compound 7 [95] and NSC76027 [94].

Virtual screening was conducted sequentially to identify in silico hits against *M. tuberculosis* topoisomerase I for assays of enzyme inhibition. The Asinex elite library of 104,000 compounds was first docked against the crystal structure (Protein Data Bank (PDB) 5D5H) of the truncated apo-enzyme of *M. tuberculosis* topoisomerase I, consisting of N-terminal domains D1–D4 plus *C*-terminal domain D5. The top 1000 hits were then docked against 1000 structures of 5D5H generated by molecular-dynamics to allow deeper binding of the molecules in the DNA-binding pockets. Six of the 82 in silico hits that were found to show inhibition of *M. tuberculosis* topoisomerase I relaxation activity share a common piperidine amide structural motif in the center that appears to interact with conserved R167 and E115 residues. Docking was then conducted for 200 compounds from the ChemBridge library that have this piperidine amide motif. Among the 96 top-scoring ChemBridge compounds purchased for testing, 18 compounds exhibited IC_50_ < 125 µM. Compound seven (Figure 9) has the lowest IC_50_ (2 µM), and >250-fold selectivity over inhibition of human topoisomerase I and *E. coli* gyrase. Antibacterial activity of the ChemBridge hit compounds against *M. smegmatis* measured in the presence of efflux pump inhibitor thioridazine showed higher MICs when *M. tuberculosis* topoisomerase I was overexpressed, consistent with these compounds acting as catalytic inhibitors of topoisomerase I. Further utilization of piperidine amide compounds for antimycobacterial activity would require modifications that improve penetrance and retention of the compounds in mycobacteria.

To identify candidates for bacterial topoisomerase I poison inhibitors, stabilization of the covalent intermediate formed after DNA cleavage was targeted for in silico screening [94]. The C-terminal domains of *E. coli* topoisomerase I (from full length structure PDB 4RUL) were connected to the 67 kDa N-terminal domains in covalent complex with cleaved DNA (PDB 3PX7) based on alignments of the two structures. CGenFF force field parameters were applied to create the 5′-phospotyrosine linkage between the enzyme and cleaved DNA, followed by the energy minimization of the structure. To demonstrate the utility of this minimized *E. coli* topoisomerase I covalent complex model for in silico screening, docking was conducted on 2263 molecules selected from the NCI DTP chemical library. Direct interaction between the enzyme and one of the top predicted hits (NSC76027, Figure 9) was confirmed with surface plasmon resonance (dissociation constant K_D_ = ~80 nM). The IC_50_ value of NSC76027 against *E. coli* topoisomerase I was estimated to be 2.2 µM [94], with a similar IC_50_ value of ~4 µM for inhibition of *M. tuberculosis* topoisomerase I relaxation activity (to be published), NSC76027 has no detectable antibacterial activity against *E. coli* MG1655 but was found to inhibit growth of *M. tuberculosis* (MIC = 35 µM) [94]. Further experiments are needed to determine the mechanism of inhibition and selectivity of NSC76027.

### 4.9. DNA Molecules as Bacterial Topoisomerase I Inhibitors

A distinct approach to target bacterial topoisomerase I is by using DNA molecules as specific inhibitors. The first step in the catalytic cycle of the enzyme is the recognition of the single-stranded regions in duplex DNA [139]. A series of 84 bp double-stranded oligonucleotides with variations in the number of bulge bases in a single-stranded loop were synthesized and tested for inhibition of the relaxation activity of *E. coli* topoisomerase I [140]. Inhibition efficiency improves as the size of the single-stranded loop increases from 1 to 10 base in length with IC_50_ reaching 63.1 nM. Inhibition of eukaryotic calf thymus topoisomerase I relaxation activity was not observed. It was hypothesized that because the single-stranded loops in the bulge oligonucleotides are not base-paired, the bulge oligonucleotides may be acting as irreversible competitive inhibitors [140].

In a second study, small circular DNA molecules with curvature that can be recognized by bacterial topoisomerase I were also investigated as competitive catalytic inhibitors [141]. Several duplex small circular oligodeoxyribonucleotides (cODNs) of varying sizes were designed, synthesized, and evaluated for inhibition of *E. coli* topoisomerase I relaxation activity. The sizes of the DNA circles synthesized mattered because the degree of bending and torsional stress in the DNA molecule increases with decreasing ring size, and the bending and torsional stress can be recognized by the enzyme. The biochemical evaluation showed inhibition of DNA relaxation activity with a IC_50_ value of 36.4 nM for the 66 bp cODN. Small single-stranded DNA circles or cODNs with seven base single-stranded mismatches have even lower IC_50_s (~10 nM) [141]. The cODNs have greater thermal stability than the bulge oligonucleotides.

A general advantage of DNA molecules is the low toxicity for the host compared to conventional drugs. The major drawback is that the ability of DNA molecules to penetrate the bacterial cell wall is very low, so more advancements in drug delivery research is needed [140,141].

## 5. Future Perspectives

Type IA topoisomerases are novel targets for infectious diseases for which no clinically approved drug has been developed so far. Effect of overexpression of the target topoisomerase should be informative on the mechanism of inhibition, overexpression of the target topoisomerase would increase the lethality of poison inhibitors. Conversely, the increased level of the target topoisomerase would help to overcome the effect of the catalytic inhibitors. The homeostatic regulation of bacterial topoisomerase I promoter activity by DNA supercoiling could increase the level of topoisomerase I expression in response to a catalytic inhibitor. The goal of finding and developing a successful drug targeting this class of topoisomerases would be greatly facilitated by the availability of the three-dimensional structure of topoisomerase–inhibitor complex, or ternary complex with DNA also present. Identification of inhibitors with high binding affinity from specific interactions with the topoisomerase protein residues would enable such structural studies. The structural information on the molecular interactions between enzyme and inhibitor would further validate the targeting of the type IA topoisomerase and allow structure-based design/optimization of type IA topoisomerase inhibitors for the development of a new therapy in the fight against the antimicrobial resistance. Determining the structure of a topoisomerase–RNA complex may also be useful for utilizing human TOP3B as a host target for providing options of antiviral therapy during flaviviruses and the coronavirus pandemics [49].

## Figures and Tables

**Table 1:** Recently described small molecule inhibitors of bacterial topoisomerase I activity.

Inhibitor	IC_50_ *E. coli* Topo I	IC_50_ *M. tuberculosis* Topo I
Bis-benzimidazole DPA 154 [110]	6.6 µM	n.d. ^1^
Bis-benzimidazole PPEF [111]	9.4 µM	n.d.
Imipramine [96]	No inhibition at 25 µM	<0.1 µM
Norclomipramine [96]	No inhibition at 25 µM	<0.1 µM
Polyamine 2471-12 [112]	7.5 µM	7.5 µM
Polyamine 2471-24 [112]	10 µM	7.5 µM
Gold(III) macrocycle 10 [113]	5 µM	10 µM
Gold(III) chelate 14 [113]	1.3 µM	1.3 µM
Fluoroquinophenoxazine 11a [114]	0.48 µM	0.98 µM
Fluoroquinophenoxazine 11g [114,115]	0.48 µM	0.24 µM
VCC891909 [98]	n.d.	<7.5 µM
Seconeolitsine (SCN) [116]	n.d.	5.6 µM
*N*-methyl-seconeolitsine (*N*-SCN) [116]	n.d.	8.4 µM
Piperidine amide 7 [95]	15.6–31.3 µM	2 µM
NSC76027 [94]	2.2 µM	4 µM

^1^ n.d.: Not determined.

## Data Availability

No new data were created or analyzed in this study. Data sharing is not applicable to this article.

## Abbreviation

WHO	World Health Organization.
PDB	Protein Data Bank.
ELISA	enzyme-linked immunosorbent assay.
MIC	minimum inhibitory concentration: The lowest concentration of an agent that stops microbial growth.
CC_50_	the 50% cytotoxic concentration: The concentration that reduces the cellular viability by 50%.
IC_50_	the half maximal inhibitory concentration: The concentration that reduces enzymatic activity by 50%.
FDA	Food and Drug Administration
NCI	National Cancer Institute
SI	selectivity index: A ratio of cytotoxicity to antimicrobial activity.
SAR	structure–activity relationship: The relationship between the chemical structure of a molecule and its biological activities.
3D-QSAR	three-dimensional quantitative structure–activity relationship: A quantification of SAR via mathematical relationships between physicochemical properties of a molecule and its biological activities.
CoMFA	comparative molecular field analysis: One of the QSAR methods, where mathematical relationships between steric and electrostatic properties of a ligand and the resulting receptor–ligand interactions are determined.
CGenFF	CHARMM General Force Field
DTP	Developmental Therapeutics Program
